# Harnessing the Power of AI for Enhanced Diagnosis and Treatment of Hepatocellular Carcinoma

**DOI:** 10.5152/tjg.2024.24325

**Published:** 2024-12-16

**Authors:** Ting Jin, Mengchen Luo, Feng Chen, Jin Bai, Jin Ding

**Affiliations:** 1 Department of Gastroenterology, Affiliated Jinhua Hospital, Zhejiang University School of Medicine, Zhejiang, China; 2 Cancer Institute, Xuzhou Medical University, Jiangsu, China

**Keywords:** Artificial intelligence, hepatocellular carcinoma, diagnosis, internet medicine, treatment

## Abstract

Since its advent, artificial intelligence (AI) has been continuously researched, and substantial progress has been made in many fields, such as the diagnosis and therapies for cancer. Due to the advantages of high efficiency, rapidity, and precision, AI has been increasingly adopted in the medical field to improve patient prognosis and the efficiency of medical procedures. Thus, AI technology has become a powerful driving force and support mechanism in the medical and health industry. Hepatocellular carcinoma (HCC) is the sixth most common cancer and the fourth leading cause of cancer mortality worldwide, with a 5-year relative survival rate of 18%. The early diagnosis and postsurgical treatment of HCC are key factors related to its prognosis, which provides an opportunity for the application of AI in HCC diagnosis and treatment. This review will summarize the application of AI in the diagnosis and treatment of HCC to provide prospects for deeper and wider applications.

**Main Points**

- Artificial intelligence plays a key role in cancer diagnosis and treatment, especially in complex data analysis.
- Artificial intelligence is also crucial for hepatocellular carcinoma (HCC) treatment.
- Its application in healthcare requires multidisciplinary collaboration and algorithm integration.
- This review helps explain AI’s role in medicine and the development of internet healthcare.

## Introduction

Artificial intelligence (AI) is a branch of computer science that studies how to realize human intelligence on machines (computers).^1^ In the past, heavy scientific and engineering calculations were mainly performed by the human brain. Currently, AI can perform these calculations faster and more accurately, significantly improving the calculation efficiency in many fields, especially in data-, knowledge-, and mental-labor-intensive fields.^2^ Medicine, as a data-intensive field, presents AI technologies with challenges and, hence, extraordinary opportunities. Artificial intelligence has attracted more attention to assist clinicians in solving medical problems due to improvements in computing power, breakthroughs in algorithm research, and the accumulation of a large amount of data, laying the foundation for application in medicine. Although algorithms are not perfect, their low misdiagnosis and low missed diagnosis rates can already be comparable to the diagnosis level of excellent doctors. In fact, AI is not a simple substitute for doctors; it provides great assistance to clinicians, releasing them from heavy low-tech labor, improving their professional skills, and allowing them to focus on providing better service to patients.^3^ Artificial intelligence has begun to change how we deliver healthcare, further making life-or-death differences in outcomes for individual patients, which is especially critical for understudied cancers such as hepatocellular carcinoma (HCC).

The incidence of HCC is increasing worldwide and is characterized by high morbidity, high malignancy, low resection, and high mortality rates.^4^ Among the various tumors of the digestive system, the precursor of HCC is still largely a pathological diagnosis often neglected by clinicians; similarly, patients with precursor lesions to HCC are also overlooked by many HCC practice guidelines from major hepatology societies.^5^ Therefore, when HCC is diagnosed, most patients are in the middle to late stages, which is mainly due to the rapid progression and the complex etiology of the disease and the lack of accurate diagnostic markers or tools for early-stage HCC.^6^ Furthermore, while multiple therapies have shown efficacy in HCC, the treatment of HCC lacks an efficient indication that guides the choice and sequence of existing therapies, leading to poor prognosis accordingly. The high mortality rate and poor prognosis of HCC make it a great threat to the social economy and public health. In theory, the diagnosis, treatment, and prognosis of HCC normally follow clinical guidelines. However, in reality, they also rely on the clinical experience and judgment of the frontline clinician. Thus, for primary hospitals and young doctors, a lack of experience in diagnosis and treatment directly affects their ability to make the best decisions and judgments regarding HCC, which is partially due to the poor prognosis of patients. Therefore, this situation offers a great opportunity for the application of AI in the diagnosis and treatment of HCC.

Although the application of AI in HCC still lacks systematic development and deeper research, the diagnosis and treatment of HCC combined with AI have, to some extent, achieved better patient prognosis. However, this field lacks comprehensive experts who are familiar with clinical knowledge and understand AI technology. Thus, we review the application of AI in the diagnosis and treatment of HCC and provide prospects for the wider application of AI in HCC.

## The Application of AI in HCC Diagnosis

Currently, the prognosis of HCC is largely dependent on the stage at which the tumor is detected, with complete curative treatment of early-stage HCC being the most effective way to improve long-term patient survival.^7^ By increasing clinical practice and investigation, the potential application of “machine learning,” “deep learning,” and “AI” has been employed to support doctors in making critical decisions related to HCC diagnosis and helping radiologists and hepatologists improve the precise diagnosis of HCC and subsequently design personalized treatments. Noninvasive imaging modalities, including ultrasound (US), computed tomography (CT), and magnetic resonance imaging (MRI), are currently considered the main applications of AI technology for the visualization of liver diseases and have important roles in HCC diagnosis.^8^ Moreover, other new diagnostic technologies combined with AI also show promise by providing further biological information on patients with HCC, enabling personalized decisions in the era of precision medicine.

### The Application of AI in Detecting Liver Nodules and Fibrosis

Cirrhosis patients are at a very high risk of developing HCC, which is a primary liver malignancy whose progression can be slowed if detected at an early stage before irreversible damage, so early detection of cirrhosis may also help in controlling disease progression toward HCC. The computer-aided diagnosis (CAD) system first selects all the candidate nodules and then classifies them as nodules/non-nodules and removes non-nodules, reducing the false positive rate. To date, CAD systems can help classify chronic liver disease (CLD) using Shear wave elastography (SWE) through a stiffness value-clustering and machine learning algorithm, achieving a sensitivity of 93.5%.^9^ Furthermore, according to reports, a CAD system combined with CT images can detect liver abnormalities from healthy liver parenchyma, such as cysts, hepatic fibrosis, and dysplastic nodules.^10^ However, expert visual assessment in cirrhotic patients with liver nodules using the current standard cannot accurately differentiate HCC from other diagnosis. The detection of candidate nodules requires high sensitivity, and as many suspicious nodules as possible should be selected. Early detection of cirrhosis with a CAD system may slow the progression of disease toward HCC quickly and efficiently.^11^ Furthermore, a study demonstrated that a radiomics machine can be trained to diagnose HCC in cirrhotic patients with indeterminate liver nodules using the European Association for the Study of the Liver guidelines.^12^

To date, many deep learning algorithms for staging liver fibrosis have been greatly developed. Yasaka et al reported sequential deep learning algorithms to diagnose liver cirrhosis through US images, which first analyze the images to detect liver capsules by using a sliding window detector, extract characteristics from each image patch using a convolutional neural network (CNN) algorithm, and finally classify an image as indicative proof of cirrhosis or not by using a support vector machine (SVM).^13^ In addition, Choi et al^14^ proposed another algorithm for fully automated staging of liver fibrosis on CT images in the portal venous phase. A recent study reported another algorithm with higher accuracy (area under the curve (AUCs), 0.97-0.98) by using cropped 2D SWE images for liver fibrosis staging compared with the measurement results of liver cirrhosis.^15^

### Liquid Biopsy Combined with AI

Liquid biopsy can be used to verify early screening results, metastasis, progression, and prognosis evaluation of HCC, which is of great value for clinical application.^16^ Artificial neural network (ANN) and decision tree data-mining methods employed to analyze profiling data, such as heat shock 70 kDa protein 8 isoform 2, cytochrome b5, and cathepsin B, were involved in identifying HCC biomarkers.^17^ Gene expression programming (GEP) is a novel algorithm with coefficients of determination of 85.37% and 86.28% on test and training sets, respectively, which shows a better outcome compared to the results from a support vector machine, a multilayer perceptron, and an ANN.^18^ Multifactor analysis methods can significantly enhance diagnostic value.^19^ Xie et al^20^ developed an expression detection system for 9 genes, including GPC3, HGF, ANXA1, FOS, SPAG9, HSPA1B, CXCR4, PFN1, and CALR, utilizing the GeXP system. In this model, specificity acts as the objective function and final diagnostic criterion. The findings indicate that the ANN detection system is the most effective for diagnosing HCC, achieving a high AUC of 0.94, sensitivity of 98%, and specificity of 85%. This shows the high sensitivity and accuracy of AI when combining serum markers.^21^ Nowadays, there are some platforms that use AI algorithms to play a role in liquid biopsies. Freenome can analyze a large number of blood samples, analyze and sequence a variety of substances, and use the in-depth learning of AI to analyze the differences in genomes, proteins, and epigenetic traits. The platform integrates complex datasets to find hidden associations that cannot be found by traditional detection methods to achieve the diagnosis and prediction of HCC. In addition, FoundationOne is an AI and next-generation sequencing-based in vitro diagnostic device based on detecting cancer genes. It can detect differences in replacement, insertion, and deletion and changes in the number of copies. To date, FoundationOne Liquid has analyzed 70 known genes, including the homologous recombination defect gene, which promotes the growth of tumor cells, including hepatoma cells.^22^ An alternative method, analyzing time-series data based on dynamic networks (ATSD-DN), is applied to provide identification for early risk identification. Importantly, ATSD-DN can measure changes in feature ratios with the nonoverlapping ratio during the development of a disease and construct dynamic networks in a systematic time dimension. A ratio of lysophosphatidylcholine (LPC) 18 : 1/free fatty acid (FFA) 20 : 5 was identified as a potential biomarker for HCC. Analyzing time-series data based on dynamic networks has shown its potential for a more complete presentation of time-series changes and effective extraction of early warning information.^23^

In addition, liquid biopsy can diagnose HCC by detecting tumor information. However, it is difficult to effectively screen all tumor cells in the blood because of the transformation ability of tumor cells.^24^ Thus, support from AI in the early diagnosis and screening by liquid biopsy is needed to solve the problem. A project from the Southern University of Science and Technology and Apostle in Silicon Valley in the United States, called “nanomagnetic beads + AI,” can enrich over 95% of the very small fragments of nucleic acids to capture rare tumor DNA. However, the screening of different tumor gene mutations depends on AI. Thus, they set up a large database containing genomic information of 35 000 cancer patients, which covers more than 25 types of cancer, including HCC. In addition, they also designed a special computer algorithm to identify effective mutations of tumors efficiently, rapidly and precisely, distinguish the suspected mutations, and discard invalid mutations.

### Segmentation and Classification of HCC Combined with AI

The widespread use of AI in the detection, segmentation, and classification of liver cancer images indicates promising performance for the automatic segmentation of liver tissues with reliably estimated necrosis rates, which can be regarded as an important imaging biomarker of clinical outcomes. Deep learning, a branch of AI, is widely applied in the medical imaging field and can detect cancer, differentiate cancer from other types of lesions, or determine the stage of a lesion. For example, Vorontsov et al^25^ proposed the use of a deep learning algorithm to automatically diagnose and segment malignant liver tumors on CT images. The automatic segmentation of healthy and cancerous liver tissues on both single-phase and multiphase CT images using a deep learning approach is more precise because each specific tissue has a specialized network.^26^ An automated algorithm for segmenting the abdomen from CT has been developed to quantify body composition, and its segmentation ability met or exceeded that of expert manual segmentation.^27^ In addition, a multichannel fusion 3-dimensional convolutional neural network (MCF-3DCNN) based on dynamic contrast-enhanced magnetic resonance images (DCE-MRI) was applied to evaluate diagnostic performance in the differentiation of the pathologic grades of HCC.^28^

Classification has an important effect on HCC diagnostics in focal liver lesions. Lesion types are often classified via abdominal MRI examination, including T2-weighted and DCE images. T2-weighted images are used for the automatic classification of focal liver lesions, and additional MRI sequences are exploited to improve the results of automatic classification and increase its usefulness in clinical aid for radiologists.^29^ A CAD system was developed to discriminate HCC with a neural network classifier and has been used in the classification of rat liver lesions; this method achieved an accuracy of 91.67%.^30^ In addition, HCC can be effectively graded based on deep features derived from diffusion-weighted imaging with multiple *b*-values using a CNN.^31^ A developed and validated proof-of-concept CNN-based deep learning system that classifies common hepatic lesions on multiphasic MRI has been applied for the precise diagnosis of HCC.^32^ Moreover, a novel morphometric measurement—the fractal dimension (FD)—has been used in an ANN with hematoxylin staining for cell nuclei and CD31/34 immunostaining for vascular elements; this method can diagnose HCC by classifying tumors as malignant or benign by analyzing captured digital images with an ANN.^33^ Rhyou developed a fully automated model for predicting liver pathological degeneration using 3 deep learning neural networks. This model employs transfer learning for the semantic segmentation of the liver and kidneys. The neural network is utilized for semantic segmentation, wherein the liver and kidney (LK) region, typically situated in the vicinity of the liver, is extracted from the ultrasound image to assess the severity of liver disease. The experimental results demonstrated comparability to those of medical experts, achieving a sensitivity of 99.8%, a diagnostic accuracy of 99.91%, and a specificity of 100%.^34^ Importantly, it is valuable and practical that noninvasive modalities can provide an alternative method to invasive liver biopsy and make a precise diagnosis through HCC classification.^8^

### Computer-Aided Diagnosis Algorithm Platform

Analysis of medical images is a time sink for radiologists, but this step is crucial for diagnosing HCC. Currently, “image recognition + AI,” as one of the hot modes, can transform many objective medical data into precise numbers, which is helpful for AI assessment and analysis of the type of liver mass and recognition of specific features with optimum accuracy in cancer detection.^35^ Into this popular step, Arterys, which, in 2017, became the first healthcare company to receive clearance from the US Food and Drug Administration for cloud-based clinical solutions that use deep learning. Furthermore, Wuhan Central Hospital recently cooperated with Tencent and the AI auxiliary diagnosis system MiYing to implement an early warning mode of abnormal image results before reporting the diagnosis to improve diagnostic accuracy and reduce the number of missed diagnoses. The system has 3 steps: an image is uploaded to the server through the network after inspection; the server calculates and recognizes the image through the set algorithm, gives the judgment result, and gives an early warning of the abnormal result to the doctor in real time; and the doctor views the AI diagnosis result to check and make a clear diagnosis and deliver a report. At present, the AI diagnosis of lung cancer and pulmonary nodules through CT has been carried out in clinical trials. After a period of time and the comparison of the diagnosis results of nearly 500 cases, the accuracy rate of the AI system reached more than 90%, which helps doctors effectively reduce the number of missed diagnoses. MiYing is being actively explored in the field of HCC diagnosis. Cem Simsek’s team used LightGBM as the primary machine learning algorithm to predict the overall survival rate of patients with HCC. The model’s AUC scores at various time points are as follows: 6 months (0.92), 1 year (0.81), 2 years (0.78), 3 years (0.81), 5 years (0.82), and 10 years (0.66). These results indicate that the model demonstrates superior performance in short-term predictions compared to long-term forecasts. Consequently, it can effectively assist physicians in evaluating the prognosis of HCC patients and formulating personalized treatment plans.^36^

## The Application of AI in HCC Treatment

In recent years, the rapid commercialization of AI products, which are widely recognized in the medical field, has brought a new treatment model. We will rely on a new generation of user-friendly, real-time big data analysis and AI and machine learning tools to provide patients with more precise and personalized treatment.

### Surgery Combined with AI

Artificial intelligence surgical robots include 4 technologies: robot technology, visual technology, instruments, and data analysis. Robots will be increasingly used to assist surgeons during surgery and will even complete some surgeries independently.^37^ The da Vinci robot assistant system, developed by the American Intuitive Surgical Company, is the most representative visual navigation system. Doctors can control the operating arm with a binocular endoscopic system through system operation. Through different parallax images and the real 3-dimensional sense of both eyes, they can better perceive the 3-dimensional distributions of intraperitoneal tissues, making the surgical field of vision clearer and increasing surgical efficiency. At present, the number of units in use in the world has exceeded 5000, and many HCC patients have benefited from da Vinci surgical robots.^38^ According to preliminary statistics, it can be seen that the application of the da Vinci Surgical System in liver tumor resection is safe and reliable, which plays an important role in improving the quality of life of patients after operation, accordingly realizing the minimally invasive operation of complex surgery.

However, a new type of robot called smart tissue anastomosis robot (STAR) has recently been developed, which makes the vision of autonomous operation a reality. Smart tissue anastomosis robot is a robot system that integrates sewing tools, robot arms, force sensors, and cameras into the hardware and software. Moreover, it has the ability to sew soft tissues, and doctors only need to monitor the robot while it is working. Researchers compared it with the operation of the da Vinci Surgical System by surgeons, and the results showed that in plane suturing, the STAR was 5 times faster than the surgeons using the da Vinci Surgical System, 9 times faster than manual laparoscopic tools, and 4 times faster than the surgeons using manual Endo 360°. In addition, experiments have also shown that stitches sewn by STAR are more precise than those sewn by surgeons.^39^

In the future, AI surgical robots will still need to realize self-learning. For example, a robot needs to reconstruct the point cloud image of a diseased area according to 3D vision, be trained on a large number of point cloud images by using a CNN, and finally perform the operation on the patient through the comprehensive technology of image feature matching and multisensory data fusion.

### Radiotherapy Combined with AI

To date, researchers have been improving AI equipment and technology for radiotherapy,^40^ especially the application of respiratory tracking technology. The tumor location can be tracked in real time, and the treatment error can reach the sub-millimeter level, which greatly improves the accuracy of radiation therapy. Combined with stereotactic, respiratory tracking, and image guidance, the AI product called CyberKnife has become the main treatment technology for HCC radiotherapy. A study showed that the incidence of adverse reactions was low in the patients who received CyberKnife therapy, and there were no serious acute hepatic reactions after radiotherapy. The treatment was well tolerated by the patients, showing a high disease control rate.^41^

However, the shortage of radiotherapy personnel and equipment is still a common problem in radiotherapy. One problem is that the sketch of the tumor radiotherapy target area and vulnerable organs not only requires doctors with good technical knowledge of tumor radiotherapy, but it also requires much repetitive work, which takes up much time and energy.^42^ On the premise of speed, accuracy, and adaptability of the sketch, if an efficient model of the tumor target area and vulnerable organ sketch can be established, the working efficiency of doctors can be improved effectively. At present, there are many atlas-based automatic target-sketching products on the market. To help oncologists make radiotherapy plans, Siemens Healthineers developed the syngo.via RT Image Suite, a software tool that uses AI to automatically draw contours of organs. By using Sherlock supercomputer training with more than 450 000 images, the AI model saves radiologists time and simplifies the task of outlining organs. Moreover, Pinnacle^3^ can automatically adapt to the patient’s organs, locate the target, outline the target area, determine the treatment target, and finally calculate and verify the plan based on model based segmentation and direct machine parameter optimization technology, which has been used in many hospitals. Recently, Google, together with the National Health Service, developed a set of AI target-sketching systems that can automatically sketch tumor areas through machine learning.^43^ RapidPlan, an AI-based commercial radiotherapy planning system from Varian, applies machine learning technology to radiotherapy planning design and is more efficient than artificial design. An intelligent radiotherapy planning design based on a planning database and planning dosimeter prediction model can be realized. Thus, AI can compensate for a lack of experienced doctors, and young doctors can improve their own radiotherapy planning systems based on AI.

### Chemotherapy Combined with AI

In addition to surgical treatment and radiotherapy, chemotherapy is also an important means to treat HCC. A research group led by Professor Sylvain Martel developed a nanorobot that can deliver drugs to targeted cancer cells by sensing oxygen concentrations. The robot delivers drugs in the “anoxic area” generated by the active proliferation of cancer cells to achieve a more precise impact on cancer tumors.^44^ Recently, another research team developed a DNA nanorobot called nanorobot-Th, which can kill cancer cells by releasing procoagulant substances into the cancer tissue, interrupting its blood supply.^45^ The greatest progress made by this kind of robot is that it can significantly improve the targeting and reduce the toxicity of chemotherapy drugs on normal human tissues. Compared with current cancer-targeting drugs, future nanorobots have been regarded as the hope for curing cancer through customizing treatment schemes according to different patients.

### Thermal Ablation Combined with AI

While most people are aware that chemotherapy and surgery are common treatments for cancer, thermal ablation is a method that is often overlooked. Thermal ablation is a minimally invasive procedure that can be employed to treat early-stage tumors.^46^ However, physicians who perform this procedure typically lack effective tools for visualizing and controlling injuries during surgery, which can result in incomplete tumor resection or damage to healthy tissue. Furthermore, doctors may need to wait up to 24 hours to assess the effectiveness of the operation. To address this challenge, TechsoMed in Israel has developed the world’s first real-time monitoring system for thermal ablation, known as BioTrace. Utilizing an NVIDIA Quadro RTX 8000 GPU, BioTrace applies an algorithm to imaging data from ultrasound equipment to facilitate monitoring and analysis during the procedure. The impact of BioTrace is substantial; the clear images and real-time feedback provided by the GPU enhance the accuracy of physicians, thereby minimizing damage to surrounding healthy tissues, expediting recovery, and reducing the risk of complications. This technique enables the tracking of real-time biological responses of tissues, allowing doctors to gain a better understanding of cancer treatment outcomes.

### Personalized Treatment of HCC Combined with AI

Individualization and precision are some of the most important directions in cancer research.^47^ For the sake of personalized treatment, phenotypic personalized medicine (PPM) finds an appropriate drug combination by a quadratic phenotypic optimization platform and a suitable dosing strategy over time by using CURATE.AI, which aims to select the optimum treatment by identifying a relationship between the input stimuli and a phenotypic response based solely on the treated individual’s data from medical charts and readouts. Phenotypic personalized medicine doesn’t require much information from the patient and the individual to identify an optimum combination therapy compared with other traditional AI techniques used for the same, such as statistical metamodeling^48^ and pharmacokinetic-/pharmacodynamic-driven precision dosing.^49^ Currently, the method based on PPM outperforms nursing standards, with few side effects other than a better prognosis, to improve the quality of personal life.

An alternative approach, the AI Clinical Decision Support System for HCC, was developed by Huaxi Hospital and YIKU MED with a large amount of HCC case data accumulated over the years to assist doctors in the proper treatment of patients. The actual clinical decisions depend on the subjective judgment of the attending physician, and the AI product provides different treatment plans for HCC patients with complex backgrounds. The AI Clinical Decision Support System for HCC can automate treatment judgments based on patient data input. Through calculations, the system can provide evidence-based, personalized, multilevel, long-term and prioritized treatment options and guidelines, as well as patient prognosis. In addition, it can provide patients with similar treatment plans, drugs, and other situations to help hepatologists or clinical teams make faster and more precise decisions for treatment.

Compared to the AI Clinical Decision Support System for HCC, at this stage, Watson is a well-known diagnosis and treatment system in the field of cancer treatment. It was established by a company in the United States, International Business Machines Corporation (IBM), and the world’s top cancer treatment center, namely Memory Sloan Kettering Cancer Center.^50^ This AI product covers 13 types of cancer, including HCC. International Business Machines Corporation Watson has helped 140 000 patients worldwide according to the latest statistics. However, the dataset is from MD Anderson Cancer Center, which contains data on patients who do not have demographic characteristics consistent with patients from other countries. The AI technology developed by YIKU MED could be the standard treatment and consultation system for cancer patients in China, according to the characteristics of Chinese cancers, combined with clinical guidelines and evidence-based medical evidence. Thus, a future research direction will be to produce a system that uses advanced AI algorithms based on various kinds of patients for HCC decision support, which will help to improve the differences in the diagnosis and treatment levels of medical institutions at all levels; accordingly, individualized and authoritative diagnosis and treatment plans can be developed for various types of cancer patients.

## Conclusion

**Figure 1.**
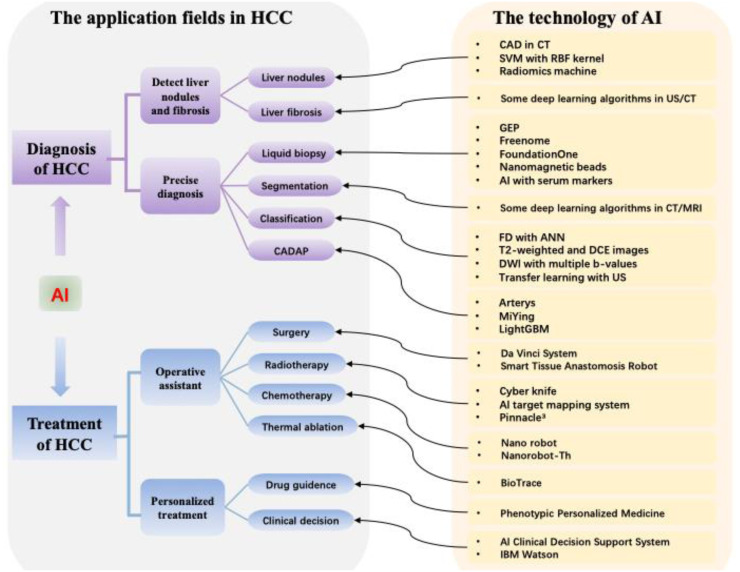
Graphical summary of AI application in diagnosis and treatment of HCC. AI, artificial intelligence; HCC, hepatocellular carcinoma; CADAP, computer-aided diagnosis algorithm platform; CAD, computer-aided diagnosis; SVM, support vector machine; CT, computed tomography; US, ultrasound; GEP, gene expression programming; MRI, magnetic resonance imaging; FD, fractal dimension; ANN, artificial neural network; DCE, dynamic contrast-enhanced; DWI, diffusion-weighted imaging.

As discussed above, with the application of AI in the area of medical and healthcare, great changes have taken place, accordingly improving the comprehensive level of hospitals. The treatment of HCC, as a medical challenge, benefits greatly from AI. The complexity and poor prognosis of liver cancer necessitate the application of AI in personalized diagnosis and treatment (Figure 1). In terms of the diagnosis of HCC, the current diagnostic tools are mainly based on the excellent ability of AI technology in image recognition and can provide increasingly more biological information for hepatologists and can assist clinicians in making more precise diagnoses. AI can also provide real-time information for patients, making diagnosis timelier and more convenient. The application of AI in the diagnosis of HCC is beneficial for the treatment of HCC patients and can be considered a bridge to guide individualized treatment decisions. For the treatment of HCC patients, novel drugs and treatments are emerging, which makes it difficult for busy clinicians to have enough time and energy to obtain, screen, and use this information (Table 1). Thus, AI plays a critical role in guiding clinicians to make individual and precise therapies. Importantly, we can establish better management of prevention, diagnosis, treatment, and rehabilitation with the assistance of AI so that clinicians can establish standardized norms in diagnosis and precise treatment. Currently, people are paying increasing attention to health problems, and the internet has increasingly been applied in the medical field. In the future, the internet + medical diagnosis and treatment models based on AI will provide people with better medical services and resources.

## Figures and Tables

**Table 1.** The AI Products Used for HCC Treatment

Task	Function	Company/Data
AI Clinical Decision Support System	Clinical decision	Huaxi Hospital & YIKU MED
IBM Watson	Clinical decision	International Business Machines & Memory Sloan Kettering Cancer Center
Phenotypic Personalized Medicine	Drug combination and dose selection	Big data & Systems biology & Omics
Da Vinci System	Operative assistance	Intuitive Surgical
Smart Tissue Anastomosis Robot	Operative assistance	Children’s National Health System
Cyber knife	Radiotherapy assistance	Stanford University Medical Centre
AI target mapping system	Radiotherapy assistance	Google DeepMind & National Health Service
Pinnacle^3^	Radiotherapy assistance	Philips
Nano robot	Chemotherapy assistance	Sylvain Martel research group
Nanorobot-Th	Chemotherapy assistance	The National Center for Nanoscience and Technology
BioTrace	Thermal ablation assistance	TechsoMed

## Data Availability

The data that support the findings of this study are available on request from the corresponding author.
